# Substance use amongst high school learners in the south of Johannesburg: Is this the new norm?

**DOI:** 10.4102/safp.v62i1.5122

**Published:** 2020-12-10

**Authors:** Debrah Mohale, Kebogile E. Mokwena

**Affiliations:** 1 Department of Public Health, Sefako Makgatho Health Sciences University, Pretoria, South Africa

**Keywords:** substance abuse, high school, risky behaviours, Johannesburg, South Africa

## Abstract

**Background:**

Substance use amongst high school learners is common in South Africa, with related risky behaviours and outcomes. Because of the social and geographical trends, studies in various parts of the country are essential to contribute to the understanding of the overall picture. The purpose of this study was to determine the prevalence of substance use, as well as the factors associated with substance use in a sample of high school learners in a suburb south of Johannesburg.

**Methods:**

A quantitative cross-sectional survey, by using a self-administered questionnaire, was conducted amongst 308 learners in four high schools. The parents of all the learners provided consent for their children to participate in the study.

**Results:**

The sample consisted of 308 learners who were in grades 8–12, with a mean age of 16.3. The majority (57%, *n* = 177) were females and 43% (*n* = 131) were males. The prevalence of substance use was 31% (*n* = 94), with 52% (*n* = 49) of those who use substances being male, compared with 48% (*n* = 45) females. Of those who used substances, 69% (*n* = 65) used alcohol, 10% (*n* = 9) smoked dagga, 5% (*n* = 5) smoked cigarettes, 7% (*n* = 7) used other substances and 46% (*n* = 43) were polyusers. Age, missing school because of illness, use of leisure time and friends using substances were significantly associated with the use of substances (*p* < 0.05).

**Conclusion:**

The prevalence of substance use was high at 31%, which is of concern because the use of legal psychoactive substances remains illegal for minors. The risky factors associated with the use of substances highlight the threat that this behaviour has on the social well-being and educational outcomes of the learners.

## Introduction

The use of psychoactive substances by adolescents at school is a major global public health problem.^1,2^ The prevalence of substance abuse has been reported to be on the rise in countries throughout the world such as Iran and Brazil and also in some African countries such as Nigeria, Ghana and Ethiopia.^3,4,5^ Because adolescence is a period of risk-taking, young people often place high value on particular behaviours that are peer approved, even if they may be risky and impact negatively on their well-being.^6^ Although the substance of choice used by learners may not be similar in all areas, there are some substances that are common at a global scale, and these include alcohol, cigarette, dagga or marijuana or cannabis and cocaine.^7^

South Africa has a high prevalence of risky alcohol use for men, women and even young people. Alcohol can be easily purchased at bottle stores, supermarkets, bars, shebeens and other unlicensed liquor outlets, which outnumber licensed ones especially in disadvantaged communities.^8^

The high prevalence of substance use in South Africa is a major concern for public health because it carries significant health risks. It also contributes to social problems such as crime and violence, with learners who use substances being more likely to experience violent acts and being involved in criminal activities.^1,3^ Learners who use substances often experience injuries in road accidents and fights, which are sometimes fatal. Substance use is also associated with risky sexual behaviours, scholastic problems like dropping out of school, and physical and mental health problems that include depressive symptoms.^7^

Substance use carries significant health risks to learners and increases both morbidity and mortality. Blood-borne infections such as hepatitis C and HIV have been reported to be common amongst people who use injectable drugs.^9^ Substance use also contributes to risky sexual behaviour because sexual inhibition is negatively affected by the effects of the substances used.^6^ They would also be more responsive to the approval they will get from their peers than they would be sensitive to the effects a hangover or the ill effects of a drug reaction that they might have. Their capacity for judgement and self-control is still not fully developed; hence their decision-making becomes risky.^6^ Substance use, therefore, contributes to high costs of health care as people using drugs need health care services as a direct result of their substance use.

Although the use and abuse of substances amongst high school learners in South Africa are acknowledged, the extent of this public health problem in various areas of the country is not known; hence this study focussed on studying this phenomenon in a specific surbub of Johannesburg.

## Methodology

### Study design

A quantitative cross-sectional survey was conducted by using a self-administered questionnaire, adapted from the 2017 National Youth Risk Behaviour Survey of the Centres for Disease Control and Prevention.

### Research setting

The study was conducted in all four high schools of a peri-urban suburb south of Johannesburg. The suburb has a population of about 71 185 people, with 66% of its population comprising of Black Africans and 33% Coloured. Afrikaans and English are the main languages used within the community.

### Recruitment

After obtaining permission from the Gauteng Province Department of Education and the management of the school, the researcher was given an opportunity to address the learners in a school hall, where she informed them about the study and requested them to participate. Those who agreed to participate were given letters to give to their parents, to request the parents to provide informed consent.

### Sample and sample size calculation

A survey of learners from grades 8–12, who were enrolled at the school during data collection, was conducted. The inclusion criterion was that parents should have provided informed consent for their children to participate.

By using the Raosoft sample size calculator with a margin of error at 5%, a confidence interval of 95% and a response distribution of 50%, a minimum sample size of 351 was calculated.

### Data collection tools

Data were collected by using an English self-administered questionnare, which was adapted from the 2017 National Youth Risk Behaviour Survey of the Centres for Disease Control and Prevention. The questionnaire had a total of 30 questions, with the first section (questions 1–10) being on socio-demographic information, and the second section (questions 11–23) on school activities and the use of substances. Section three (questions 24–30) was on the views and experiences of the learner on substance use. The learners who indicated that they did not use substances had to skip follow-up questions in this section, to complete the last three questions (28–30).

### Data collection

Learners were given consent forms prior to the day of data collection to obtain parental consent. These were collected on the day of data collection, and only learners whose parents had provided the informed consent were assembled in the school hall or classrooms. The purpose of the study was repeated, and the learners were given an opportunity to ask questions or seek clarification. Learners who were willing to participate were then provided with assent forms to sign, which was followed by the data collection questionnaire. The learners were not required to identify themselves as the questionnaire was completed anonymously, and none of the responses could be linked to any participants. The completed questionnaires were collected by the researcher as each learner exited the venue.

### Analysis

The data were captured into Microsoft Excel and exported to the software STATA version 13 for analysis. Descriptive statistics was used to determine the prevalence of substance abuse and was depicted in the form of tables, frequencies and percentages. Simple linear regression was used to explore any association between a range of socio-demographic variables and substance abuse. Lastly, a logistic regression was performed to explore the strength of the factors associated with substance abuse by computing the odds ratio at 95% confidence interval and a *p*-value <0.05 was considered statically significant.

### Ethical consideration

Ethical approval to conduct the study was obtained from the Sefako Makgatho Health Sciences University Research Ethics Committee (SMUREC) before data collection commenced. Permission to conduct the study was sought from the Department of Basic Education in Gauteng, the District Manager of the Gauteng South Department of Education and principals of the participating schools. Informed consent was sought from parents of the learners. Participation was voluntary and participants were informed that they can withdraw from the study at any time without any penalty being imposed on them (ethical clearance number: SMUREC/H/73/2017:PG).

## Results

A total of 400 questionnaires were distributed to potential participants during data collection, and 308 (77.0%) had complete data for analysis. Their ages ranged from 13 to 21, with a mean of 16.3 and a median of 16 (SD 1.5). For analysis purposes, the ages were categorised according to the developmental phases of early teens (13–15), middle teens (16–18) and early youth (19–21). As shown in Table 1, 57% of learners were females and 43% males. The participants were in grades 8–12, with the junior phase (grades 8–9) at 19.2% (*n* = 59) and the senior phase (grades 10–12) in the majority at 80.8% (*n* = 249). The majority (73%) of the learners were Black, and Christian learners were in the majority at 82.7%. Nine per cent of the parents were unemployed, 57% of fathers and 75% of mothers had acquired secondary and tertiary education. The majority (70%) had fewer than 3 children in their family.

**TABLE 1:** Descriptive socio-demographic characteristics of the sample (*n* = 308).

Characteristics	*N*	%
**Age (*n* = 306)** †		
13–15 years (early teens)	79	25.8
16–18 years (middle teens)	208	67.9
19–21 years (early youth)	19	6.2
**Gender (*n* = 308)**		
Male	131	42.5
Female	177	57.5
**Race (*n* = 293)** ‡		
Coloured	79	27
Black	214	73
**Grade (*n* = 308)**		
Gr 8–9	59	19.2
Gr 10–12	249	80.8
**Number of children in family (*n* = 306)** §		
Below 5 children	271	88.6
Above 6 children	35	11.4
**Religion (*n* = 301)** ¶		
Christians	249	82.7
Non-Christians	52	17.3
**Employment of parents (*n* = 303)** ††		
Unemployed	28	9.2
Employed	275	90.8
**Education level of father (*n* = 302)** ‡‡		
Did not complete high school	131	42.5
Completed high school	177	57.5
**Education level of mother (*n* = 308)**		
Did not complete high school	76	24.68
Completed high school	232	75.32

† , 2 missing values;

‡ , 15 missing values;

§ , 2 missing values;

¶ , 7 missing values;

†† , 5 missing values;

‡‡ , 6 missing values.

### Prevalance of substance use

The prevalence of substance use was 31% (*n* = 94), most used substances with their friends, 46% of these were polyusers and 47.2% (*n* = 51) tried to quit but were not successful.

The type of substances used is shown in Table 2.

**TABLE 2:** Types of substances used (*n* = 94).

Substance	Number	%
Cigarette smoking	5	5
Alcohol use	65	69
Dagga use	9	10
Hookah pipe	5	5
Other unknown	7	7
Polyusers	43	46

The use of substances was more prevalant in males at 52%, and alcohol, which included beers, wine, whisky and vodka, was the only substance that had a higher prevalance amongst females, at 51.%. Table 3 shows the patterns of substance use according to gender.

**TABLE 3:** Patterns of substance use according to gender.

Substance use	M (*N*)	%	F (*N*)	%	Total
Yes	49	52.13	45	47.9	94
Cigarette use	16	61.54	10	38.5	26
Alcohol	38	49.35	39	50.7	77
Dagga use	17	62.96	10	37.0	27
Hookah pipe	5	71.43	2	28.6	7

The use of substances by friends was more prevalent amongst males, with family and adult use more prevalent for female learners. Substance use according to age categories indicated a higher proportion in the 16–18 age category as indicated in Table 4.

**TABLE 4:** Substance use according to age (*n* = 94).

Age category	Substance of use		Cigarettes		Alcohol		Dagga	
	*N*	%	*N*	%	*N*	%	*N*	%
13–15 years	16	17.4	4	15.4	12	15.8	5	19.2
16–18 years	67	72.83	20	76.9	56	73.7	20	76.9
19–21 years	9	9.8	2	7.7	8	10.5	1	3.9

Table 5 indicates the proportion of risky behaviours experienced by learners who reported using substances, which include serious problems with friends, having sex without a condom and trouble with the police.

**TABLE 5:** Associated risky behaviours.

Risky behaviour	*N*	%
Serious problems with friends	26	55.3
Sex without a condom	18	38.3
Trouble with the police	3	6.4

**Total**	**47**	**100**

The majority of the learners (70%, *n* = 214) did not use psychoactive substances, and Table 6 shows the reasons stated for not using substances, with personal choice as the most cited reason.

**TABLE 6:** Reasons for not using substances (*n* = 179).

Reason for not using substances	M (*N*)	%	F (*N*)	%
Friends do not use	2	25	6	75
Parental guidance	10	47.62	11	52.4
Personal choice	43	32.33	90	67.7
Religious influence	3	30	7	70
Sporting activities	4	57.14	3	42.9

### Logistic regression results

The results of the logistic regression to explore factors that are associated with substance use are presented in Table 7. Older age, missing school because of illness, no plans for use of leisure time and friends’ use of substances were found to be significantly associated with substance use (*p* ≤ 0.05). It was not clear whether missing school was a result of substance use or learners who had used substances had indicated missing school and not being involved in any activities for leisure time.

**TABLE 7:** Factors associated with substance abuse.

Variables	Odds ratio	95% CI	*p*
Increasing age	1.455853	1.09312–1.938953	0.010
Gender	0.6993188	0.3528612–1.385947	0.305
Race	0.9268706	0.3974498–2.161504	0.860
Employment status of parents	0.6121432	0.1934235–1.938112	0.404
Religion	1.688005	0.6926964–4.113432	0.249
How do they go to school	0.5187055	0.2465102–1.091458	0.084
Miss school because of illness	4.926415	2.364184–10.26551	0.000
Miss school for other reason	1.822569	0.9051242–3.669946	0.093
Repeat class	1.070486	0.4842–2.366665	0.866
Plans for use of leisure time	6.036122	2.48169–14.68143	0.000
Friend use of substances	4.378269	2.001131–9.579201	0.000
Family member use of substances	0.8426663	0.4135963–1.716859	0.637

## Discussion

The results of the study show a high prevalence of substance use by high school learners in this sample, which included both male and female learners across all grades of high school. The results confirmed a high prevalence of substance use for both genders, with the use of alcohol, cigarettes and dagga being the most commonly used substances. Although the use of substances was found to be below 50% of the total sample, the prevalence for male use of dagga and cigarettes was high at above 50% of those that use substances, whilst female use of alcohol was 51%.

These findings were similar to a study conducted in a different area of Johannesburg, which found a higher prevalence amongst males,^10^ whilst some studies show males to be twice more likely to use substances than females.^11,12^ However, the prevalence of substance use amongst males and females in this study was almost the same, which is similar to previous studies which found a small difference between male and female substance use prevalence.^13,14^ This difference is explained by reports of increase in both the frequency and amount of consumption by females.^15^

The high prevalence of substance abuse in this sample raises public health concerns as substance use and abuse carry significant health risks^7^ for this sample of young people in school. This is because the learners, who are in the adolescence phase, are at a time of greatest risk taking and often place high value on any reward they get from taking risks, if such actions are acknowledged and approved by their peers.^6^ These risks include social problems such as accidents and injury, violence, crime, high-risk sexual behaviour and poor social relations.^11^ Academic risks include repeating a grade, missing school because of illness or other reasons, school dropout and low academic aspirations.^7^ Substance use, especially alcohol, is associated with negative impacts on school work like absenteeism, low performance, truancy and delinquency.^16^ In this sample, reported impacts were serious problems with friends, having sex without a condom and trouble with the police.

The results also confirm the significant impact of peer pressure, which is the desire by adolescents to fit in and be socially accepted by their peers because they have a high value for the opinion of their peers.^8,16,17^ In this study, the use of substances by friends was associated with substance use. This suggests that for some young people, the need for approval may surpass reasoning and compliance with other values of the family or school.

The most commonly used substances by the high school learners included alcohol, which was followed by cigarettes and dagga, and the least used substances being ecstasy and inhalants. These findings are similar to others, which found that the most commonly used substances by adolescents in South Africa include alcohol, cigarettes and dagga.^7,18,19^ The trend is similar globally, and alcohol, cigarette and dagga are amongst the most common substances of abuse by adolescents at school.^4,5,20,21^ The common use of these three substances can be explained by their ease of access, that it is more socially acceptable to use alcohol and cigarette and that dagga is easy to grow. Other studies found that alcohol is the most abused drug, with ease of access being the main contributory factor.^8,21,22,23^ This was confirmed by the results of this study, which identified alcohol as the most used substance by the majority of those who use substances. Alcohol is reported to be attractive to young people because it is seen as a sign of maturity and it is easily accessible.^24^

The results of the study supported previous reports that adolescents start using substances at an early age of between 13 and 15 (Table 4), which is similar to findings from previous studies that found the mean age of initiation to be between 13 and 14 years.^4,14,15,25^ The early initiation of substance use comes with a variety of negative health and social outcomes.^22^

The findings of this study identify the social environment as influential in substance abuse, which indicates a need to target groups rather than the individual for interventions aimed at preventing substance use. Moreover, the experiences of physical and psychological discomforts such as stress, low self-confidence and being overwhelmed by challenges experienced in their lives, which are often cited as drivers to substance use, also affect their peers, families or even society at large.^17,24^ As a social environment, on one the hand the school system influences adolescents against the use of drugs, often through the use of rules, whilst on the other hand, the influence of other learners can succeed in planting a rebellious and negative influence against the school system. It is for that reason careful and well thought out interventions should be developed in an effort to prevent substance abuse amongst learners in a school environment, which learners often use as a means to escape painful experiences of failure of various types.^24^

This study found that almost half (46.2%) of the learners who use substances tried to quit, which is similar to another study conducted in a rural area of South Africa, where it was found that learners often try but fail to quit the use of substances.^13^ This indicates that some substance using young people realise the risk of using substances but fail to quit because addiction has set in. Their attempt and failure to quit is one criterion that qualifies their use of substances to be viewed as abuse.^26^

The study found that the majority of learners who do not use substances cited personal choice and parental guidance, which is encouraging as it confirms that family values still have a role in discouraging risky behaviours. The cited reasons give an indication of areas to focus on, when interventions for prevention of substance abuse are developed and implemented.

## Limitations of the study

Although the response rate was high at 77%, 23% of the questionnaires had missing data of more than 10% each and could therefore not be included in the analysis. Their inclusion could have given a clearer picture of substance use in this sample.

## Conclusion

The use of substances by learners seems to be a new norm in South Africa, with resultant negative impacts on their physical, social and academic life. The common use of cannabis and alcohol, which is illegal for minors, indicates criminal behaviour which needs to be addressed. The study also found a higher prevalence of alcohol amongst female learners, which is increasingly being reported, and calls for more studies to investigate contributory factors to this phenomenon as well as identify protective factors within the communities to counteract this behaviour.

### Recommendations

Successful interventions to prevent and/or counteract substance abuse in schools need to be strengthened, which will improve the teaching and learning environment, and thus enhance successful educational outcomes. It is also recommended that schools make efforts to assist students to plan for the use of their leisure time, which may include encouragement to participate in sports at school. Lastly, it is recommended that the reasons cited for the non-use of substances be explored further to use them as the foundation of building resilience against substance use and abuse.
